# Protective Effect of Fermented Sea Tangle Extract on Skin Cell Damage Caused by Particulate Matter

**DOI:** 10.7150/ijms.93034

**Published:** 2024-03-31

**Authors:** Mei Jing Piao, Kyoung Ah Kang, Pincha Devage Sameera Madushan Fernando, Herath Mudiyanselage Udari Lakmini Herath, Young Sang Koh, Hee Kyoung Kang, Yung Hyun Choi, Jin Won Hyun

**Affiliations:** 1 College of Medicine, and Jeju Research Center for Natural Medicine, Jeju National University, Jeju 63243, Republic of Korea.; 2 College of Oriental Medicine, Dongeui University, Busan 47340, Republic of Korea.

**Keywords:** fermented sea tangle extract, particulate matter 2.5, oxidative stress, apoptosis

## Abstract

The skin is directly exposed to atmospheric pollutants, especially particulate matter 2.5 (PM_2.5_) in the air, which poses significant harm to skin health. However, limited research has been performed to identify molecules that can confer resistance to such substances. Herein, we analyzed the effect of fermented sea tangle (FST) extract on PM_2.5_-induced human HaCaT keratinocyte damage. Results showed that FST extract, at concentrations less than 800 μg/mL, exhibited non-significant toxicity to cells and concentration-dependent inhibition of PM_2.5_-induced reactive oxygen species (ROS) production. PM_2.5_ induced oxidative stress by stimulating ROS, resulting in DNA damage, lipid peroxidation, and protein carbonylation, which were inhibited by the FST extract. FST extract significantly suppressed the increase in calcium level and apoptosis caused by PM_2.5_ treatment and significantly restored the reduced cell viability. Mitochondrial membrane depolarization occurred due to PM_2.5_ treatment, however, FST extract recovered mitochondrial membrane polarization. PM_2.5_ inhibited the expression of the anti-apoptotic protein Bcl-2, and induced the expression of pro-apoptotic proteins Bax and Bim, the apoptosis initiator caspase-9, as well as the executor caspase-3, however, FST extract effectively protected the changes in the levels of these proteins caused by PM_2.5_. Interestingly, pan-caspase inhibitor Z-VAD-FMK treatment enhanced the anti-apoptotic effect of FST extract in PM_2.5_-treated cells. Our results indicate that FST extract prevents PM_2.5_-induced cell damage via inhibition of mitochondria-mediated apoptosis in human keratinocytes. Accordingly, FST extract could be included in skin care products to protect cells against the harmful effects of PM_2.5_.

## Introduction

Air pollution poses a growing threat to human health; especially, the increase in particulate matter 2.5 (PM_2.5_), with a particle size of 2.5 microns or less, severely affects quality of life. Many studies have shown that PM_2.5_ can induce diseases such as cancer, immunosuppression, aging, and inflammation, and that the associated underlying mechanism is related to oxidative stress 1-5. The skin that is in direct contact with the external environment is divided into two layers, the epidermis and dermis. The epidermis is on the surface of the skin and is most susceptible to environmental pollution. In skin-related studies, PM_2.5_ was found to reduce lipid synthesis, promote inflammatory cytokine production in human sebocytes 6, and inhibit ciliogenesis in skin keratinocytes 7. Among various PM, polycyclic aromatic hydrocarbons and organic compounds are highly lipophilic and therefore easily penetrate the skin 8, 9. Recently, we published a study on the mechanism of oxidative damage induced by PM_2.5_ in skin epidermal cells 10, 11. Although PM_2.5_ is highly harmful, the development of protective substances is lacking.

*Laminaria japonica* (sea tangle), a large marine brown alga that grows in low-temperature seawater, is an edible seaweed. *L. japonica* has the ability to reduce blood lipids, lower blood glucose, and regulate immunity, in addition to exerting anti-coagulation, anti-cancer, detoxification, and anti-oxidation effects 12-15. *L. japonica* contains many active ingredients that are beneficial to the human body, such as essential amino acids, vitamins, minerals, essential fatty acids, and bioactive compounds 13, 16. Microbial fermentation of *L. japonica* can enrich bioactive substances such as polyphenols, flavonoids, and polysaccharides and reduce the toxicity of natural extracts. Recent studies have shown that fermented sea tangle (FST) promotes nutrient supply by reducing the molecular weight of polysaccharides and improves skin condition by increasing anti-inflammatory activity 17. The levels of some amino acids in *L. japonica*, such as aspartic acid, serine, and threonine, are reduced by fermentation with *Lactobacillus brevis*, whereas those of alanine, valine, glycine, and leucine are significantly increased. Moreover, most glutamic acid in *L. japonica* is converted to gamma-aminobutyric acid (GABA) 18. GABA-enriched FST extract not only has antioxidant effects but also prevents or treats diseases, such as cognitive disorders and liver damage 19-22. GABA improves skin moisture, skin wrinkles, and epidermal thickness on human skin cells and hairless mice 23. GABA improves skin elasticity in human dermal fibroblasts by upregulating elastin synthesis and elastin fiber formation 24. Topical application of GABA can serve as a potential remedy for skin aging by promoting the biosynthesis of type I collagen by interacting with GABA receptors in the dorsal skin of mice 25. Regardless, research on substances that could confer resistance to oxidative damage induced by PM_2.5_ was lacking. Considering the antioxidant potential of FST, this study aimed to determine whether FST extract can be developed as an effective skin-protectant that can mitigate the damaging effects of PM_2.5_, which spreads through the air.

## Materials and Methods

### Preparation of FST extract and PM_2.5_

FST extract was provided by Professor Young Hyun Choi of Dongeui University (Busan, Republic of Korea) 19. Sea tangle was added to water at a ratio of 1:15 (w/v), and yeast extract and glucose were added according to the amount of sea tangle added. A sea tangle solution was obtained by autoclaving the mixture at 121 °C for 30 min, and *Lactobacillus brevis* BJ20 (accession number KCTC 11377BP) culture solution was added at a concentration of 1.2% (v/v), mixed, and cultured at 37 °C for 2 days. The extract obtained through fermentation was filtered, freeze-dried, stored, and dissolved in Milli-Q Water to prepare a 10 mg/mL stock solution. The diesel particulate matter NIST 1650b (PM_2.5_) was purchased from Sigma-Aldrich, Inc. (St. Louis, MO, USA), and its stock solution was prepared as previously described 10.

### Reagent information

Reagent information is described in Table 1.

### Cell culture

Human HaCaT keratinocytes (Cell Lines Service GmbH, Eppelheim, Germany) were maintained in Dulbecco's Modified Eagle Medium/high glucose (Hyclone Laboratories, South Logan, UT, USA) containing 10% fetal bovine serum (Gibco, Life Technologies Co., Grand Island, NY, USA) and an antibiotic-antimycotic (Gibco) at 5% CO_2_, 37 °C, and humidified atmosphere conditions.

### Cell viability

Cells were seeded at a density of 1.0 × 10^5^ cells/mL in 24-well plates. The FST extract was administered after a 16 h incubation at a concentration of 100, 200, 400, 800, or 1,600 μg/mL. Subsequently, MTT solution was added to each well. After 4 h, dimethyl sulfoxide solution was added, and the absorbance was read at 540 nm using a microplate reader. In addition, cells were treated with 30 μM Z-VAD-FMK or/with 800 μg/mL FST extract. After 1 h, 50 μg/mL PM_2.5_ was added to the cells. A 0.1% trypan blue solution was added into the cell suspension, observed under a 20 × microscope, and photographed. Cell viability (%) = Number of unstained cells/(Number of unstained cells + Number of stained cells) × 100.

### DPPH radical scavenging assay

FST extract, at a concentration of 100, 200, 400, 800, or 1,600 μg/mL, was added to 1 × 10^-4^ M DPPH methanol solution and shaken for 3 h. The absorbance was measured at 520 nm using a spectrophotometer.

### Detection of intracellular reactive oxygen species (ROS)

Cells were seeded in a 96-well plate for detection of ROS using a fluorescence spectrophotometer (PerkinElmer, Waltham, MA, USA) or a 6-well plate for detection of ROS using flow cytometry (Becton Dickinson, Mountain View, CA, USA) at a density of 0.8 × 10^5^ cells/mL. FST extract was administered at a final concentration of 100, 200, 400, 800, and 1,600 μg/mL, and 1 mM NAC was included as the positive control. After incubation for 30 min, 1 mM H_2_O_2_ or 50 μg/mL PM_2.5_ was added to the plates, incubated for an additional 30 min at 37 °C, and 25 μM H_2_DCFDA solution was added. The fluorescence intensity (FI) of the 2',7'-dichlorofluorescein product was quantified using a fluorescence spectrophotometer or via flow cytometry. The intracellular ROS scavenging activity (%) was calculated as [(H_2_O_2_-treated optical density) - (FST extract + H_2_O_2_-treated optical density) / (optical density of H_2_O_2_ treatment)] × 100, as detected using a spectrophotometer. The intracellular ROS scavenging activity (%) was calculated as [(PM_2.5_-treated FI) - (FST extract + PM_2.5_-treated FI) / (PM_2.5_-treated FI)] × 100, as detected using a flow cytometer. For image analysis of intracellular ROS, cells were seeded at 1.0 × 10^5^ cells/mL in 4-well glass chamber slides and treated with 800 μg/mL FST extract. After 30 min, PM_2.5_ was administered to the cells. After incubation for 30 min, H_2_DCFDA was added to each well and incubated for an additional 10 min at 37 °C. Cells on the slides were fixed with a mounting medium, and cell images were acquired using a fluorescence microscope (Carl Zeiss, Jena, Germany).

### Measurement of superoxide anion and hydroxyl radical

To analyze superoxide anion, the following materials were sequentially added: PBS or 800 μg/mL FST extract, 3 M DMPO, 0.25 U/mL xanthine oxidase, and 10 mM xanthine, each at 20 μL. For hydroxyl radical analysis, the following materials were sequentially added: PBS or 800 μg/mL FST extract, 0.3 M DMPO, 10 mM FeSO_4_, and 10 mM H_2_O_2_, each at 20 μL. Following rapid mixing, the reaction was performed for 2.5 min, and signaling was recorded using an electron spin resonance (ESR) spectrometer. The ESR parameters are shown in Table 2.

### Single-cell gel electrophoresis (Alkaline comet assay)

Cells at a density of 0.5 × 10^5^ cells/mL were seeded and treated with 800 μg/mL FST for 30 min, after which 50 μg/mL PM_2.5_ was added and allowed to incubate for 30 min. 100 μL of 0.5% low melting point agarose was added and mixed at 39 °C, and 50 μL of the cell suspension was spread on a microscope slide precoated with 1% normal melting point agarose. Cell-coated slides were immersed in a lysis buffer solution (2.5 M NaCl, 100 mM Na_2_EDTA, 10 mM tris-pH 10, 1% N-lauroylsarcosinate, 1% triton X-100) at 4 °C in the dark for 1.5 h. Slides were electrophoresed for 20 min at 25 V and 300 mA, stained with ethidium bromide, and images were acquired using a fluorescence microscope equipped with image analysis software (Kinetic Imaging, Komet 5.5, UK). From each slide, the total fluorescence percent and tail length were recorded based on 50 cells.

### Western blot analysis

Cell lysates were subjected to sodium dodecyl sulfate-polyacrylamide gel electrophoresis, and the separated proteins were transferred to a nitrocellulose membrane, and subsequently incubated with phospho-histone H2A.X (Ser139), Bcl-2, Bax, Bim, caspase-3, caspase-9, PARP, and actin primary antibodies. Blots were further incubated with the secondary antibody. Protein bands were detected using the EZ-western kit (DoGenBio, Seoul, Republic of Korea).

### Protein carbonylation

Total protein from the cells was isolated using protein lysis buffer, and the protein concentration was measured with a Quant-iT™ protein assay kit (Thermo Fisher Scientific). Protein carbonylation was determined according to the instructions of the OxiSelect™ protein carbonyl ELISA kit (Cell Biolabs, San Diego, CA, USA).

### Lipid peroxidation assay

The fluorescent probe DPPP was applied to the cells at 1.0 × 10^5^ cells/mL in 4-well glass chamber slides and a cell slide was prepared. Images were captured using a confocal microscope equipped with FV10-ASW Viewer 4.2 software (Olympus, Tokyo, Japan), and fluorescence intensity was analyzed using ImageJ Version 1.47 26.

### Measurement of intracellular Ca^2+^

The cell slide was prepared after applying the fluorescent probe Fluo-4 AM to the cells. Images were captured using a confocal microscope (Olympus), and fluorescence intensity was quantified 10.

### Nuclear staining with Hoechst 33342

Hoechst 33342 fluorescent dye was added to each well and incubated for 10 min. The extent of nuclear condensation and nuclear fragmentation was assessed by fluorescence microscopy equipped with a CoolSNAP-Pro color digital camera (Media Cybernetics, Rockville, MD, USA). The number of apoptotic cells was then quantified. Apoptotic index = (Number of apoptotic cells in the treated group/Total number of cells in the treated group)/(Number of apoptotic cells in the control group/Total number of cells in the control group).

### Mitochondrial membrane potential (Δψ_m_) measurement

Cell slides were prepared after treating the cells with the lipophilic cationic fluorescent dye JC-1. Images were captured using a confocal microscope equipped with FV10-ASW Viewer 4.2. Fluorescence intensity was measured using ImageJ Version 1.47 to analyze Δψ_m_.

### Statistical analysis

Statistical significance was determined by performing an analysis of variance and the Tukey test with SigmaStat 3.5 version software (Systat Software Inc., San Jose, CA, USA). All values are expressed as the mean ± standard error. A *p* < 0.05 indicated statistical significance.

## Results

### Effect of FST extract on ROS generation

**Figure 1:**
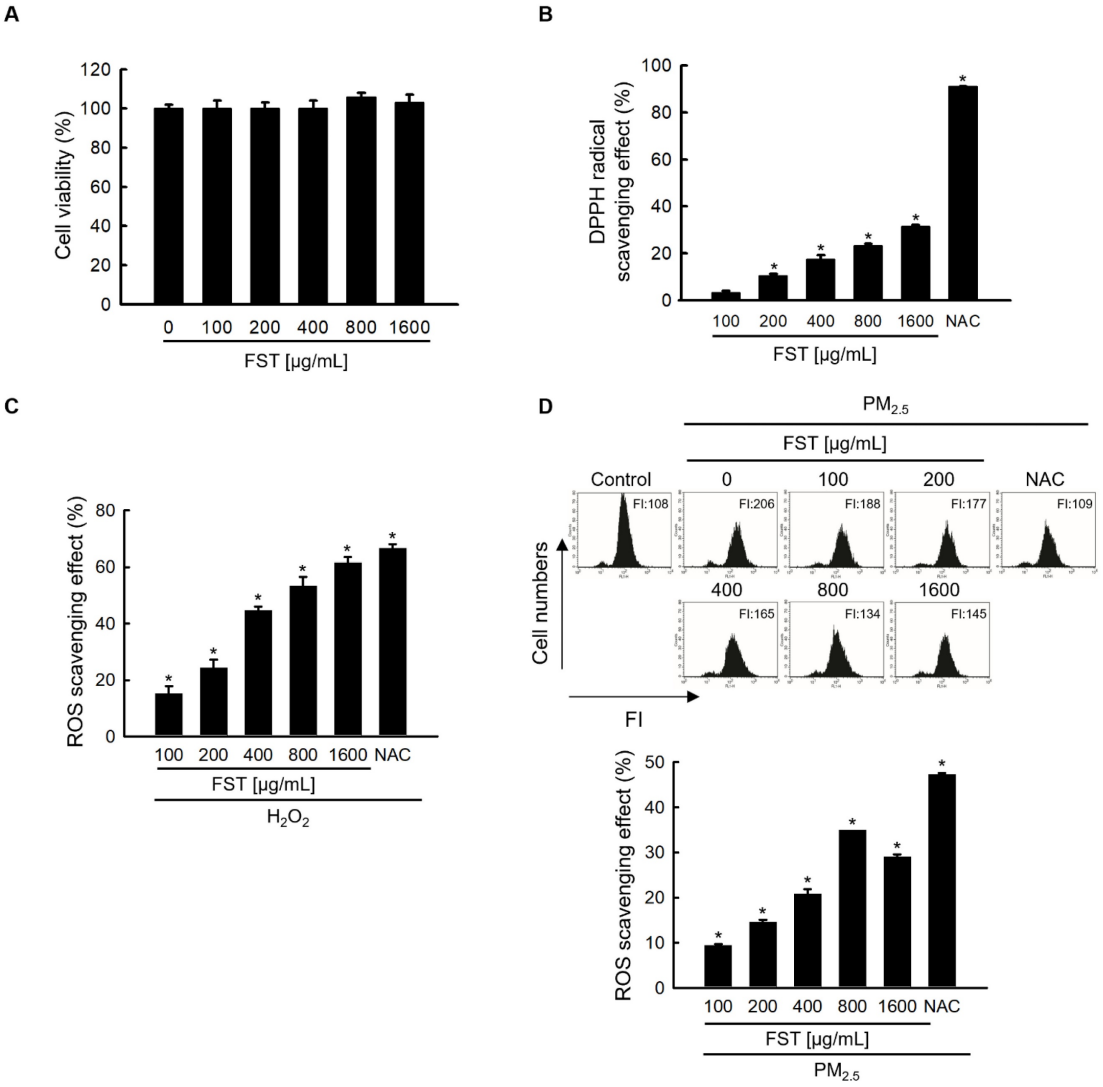

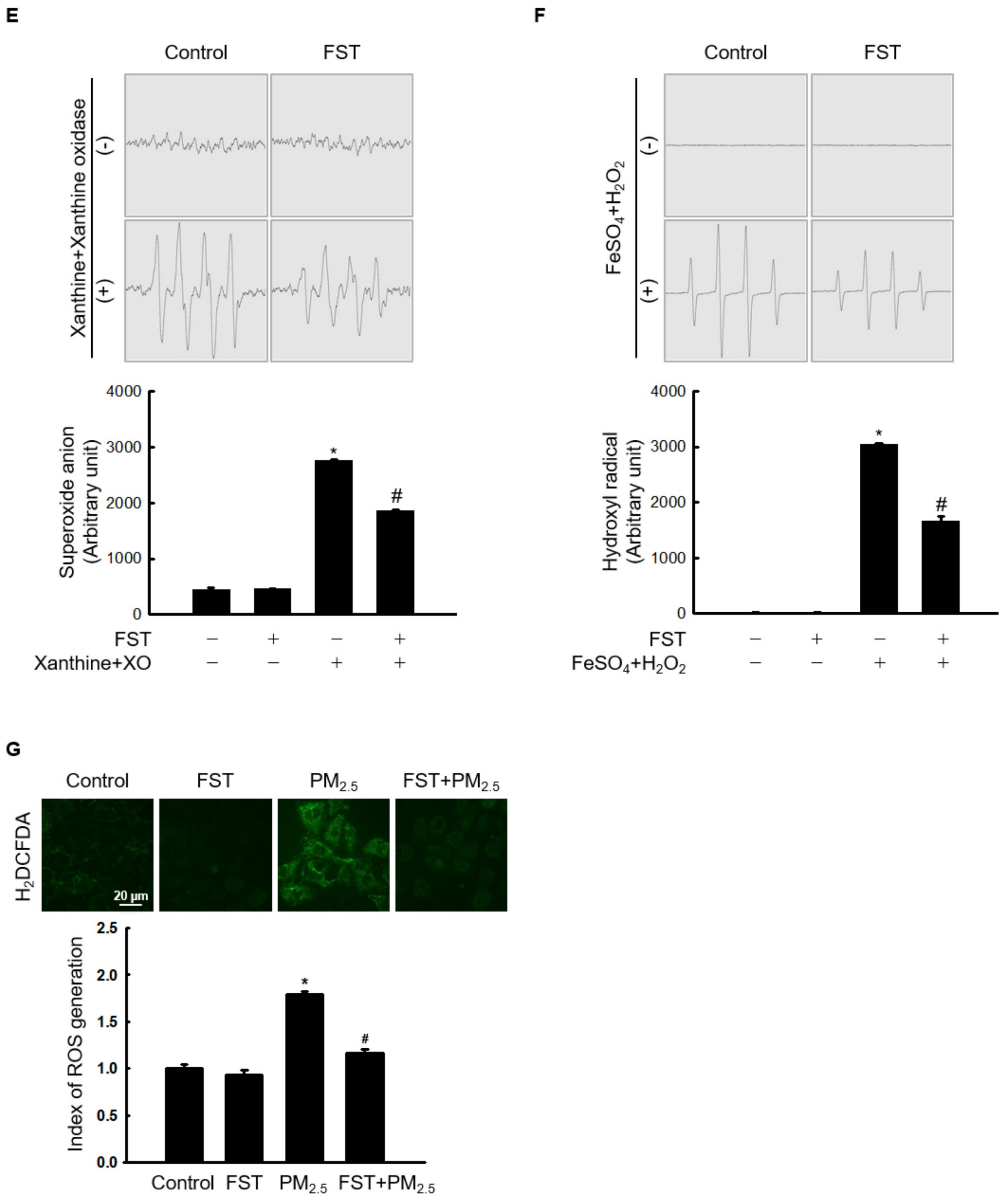
Effects of FST extract on cytotoxicity and ROS. (A) Cell viability was determined by performing MTT assay. (B) The ability to scavenge DPPH radicals was measured using a spectrophotometer at a wavelength of 520 nm. NAC was used as a positive control. ^*^ *p* < 0.05 compared to the FST extract-untreated group. (C, D) After staining with H_2_DCFDA, intracellular ROS produced from H_2_O_2_ or PM_2.5_ were detected using (C) a spectrophotometer or (D) flow cytometry. NAC was used as a positive control. ^*^ *p* < 0.05 compared to the H_2_O_2_ or PM_2.5_-treated group. (E) The superoxide anion adducts DMPO-OOH and (F) the hydroxyl radical adducts DMPO-OH were detected using an ESR spectrometer. ^*^ p < 0.05 and ^#^* p* < 0.05, compared to control and superoxide anion or hydroxyl radical group, respectively. (G) Intracellular ROS production was detected using confocal microscope after H_2_DCFDA staining. ^*^ p < 0.05 and ^#^* p* < 0.05, compared to the control group and PM_2.5_-treated group, respectively.

To determine the optimal experimental conditions for the FST extract, its cytotoxicity was first determined. MTT assay results showed non-toxicity to human HaCaT keratinocytes at concentrations less than 1,600 μg/mL (Figure 1A). The FST extract scavenged DPPH radical in a concentration-dependent manner, with a scavenging rate of 31% at the highest concentration of 1,600 μg/mL, while that of the positive control NAC (1 mM) was 91% (Figure 1B). Intracellular ROS induced by H_2_O_2_, measured using a spectrophotometer, were also eliminated in a concentration-dependent manner of FST extract. At 1,600 μg/mL of FST extract, the ROS scavenging rate was 58%, while that of the positive control NAC (1 mM) was 61% (Figure 1C). FST extract also showed a scavenging effect on PM_2.5_-induced intracellular ROS, exhibiting that the scavenging rate was the highest of 34% at 800 μg/mL, and NAC was 47% (Figure 1D). Therefore, we selected 800 μg/mL as the concentration for subsequent experiments. ESR spectroscopy showed that the superoxide anion signal generated in the xanthine/xanthine oxidase (XO) system was 2,756 but was reduced to 1,868 upon FST extract treatment (Figure 1E). The Fenton reaction produced a hydroxyl radical signal of 3,044, whereas the FST extract reduced this to 1,657 (Figure 1F). Furthermore, confocal microscopy showed that the FST extract significantly attenuated PM_2.5_-induced ROS production (Figure 1G).

### Effect of FST extract on PM_2.5_-induced oxidative damage to cellular substances

**Figure 2:**
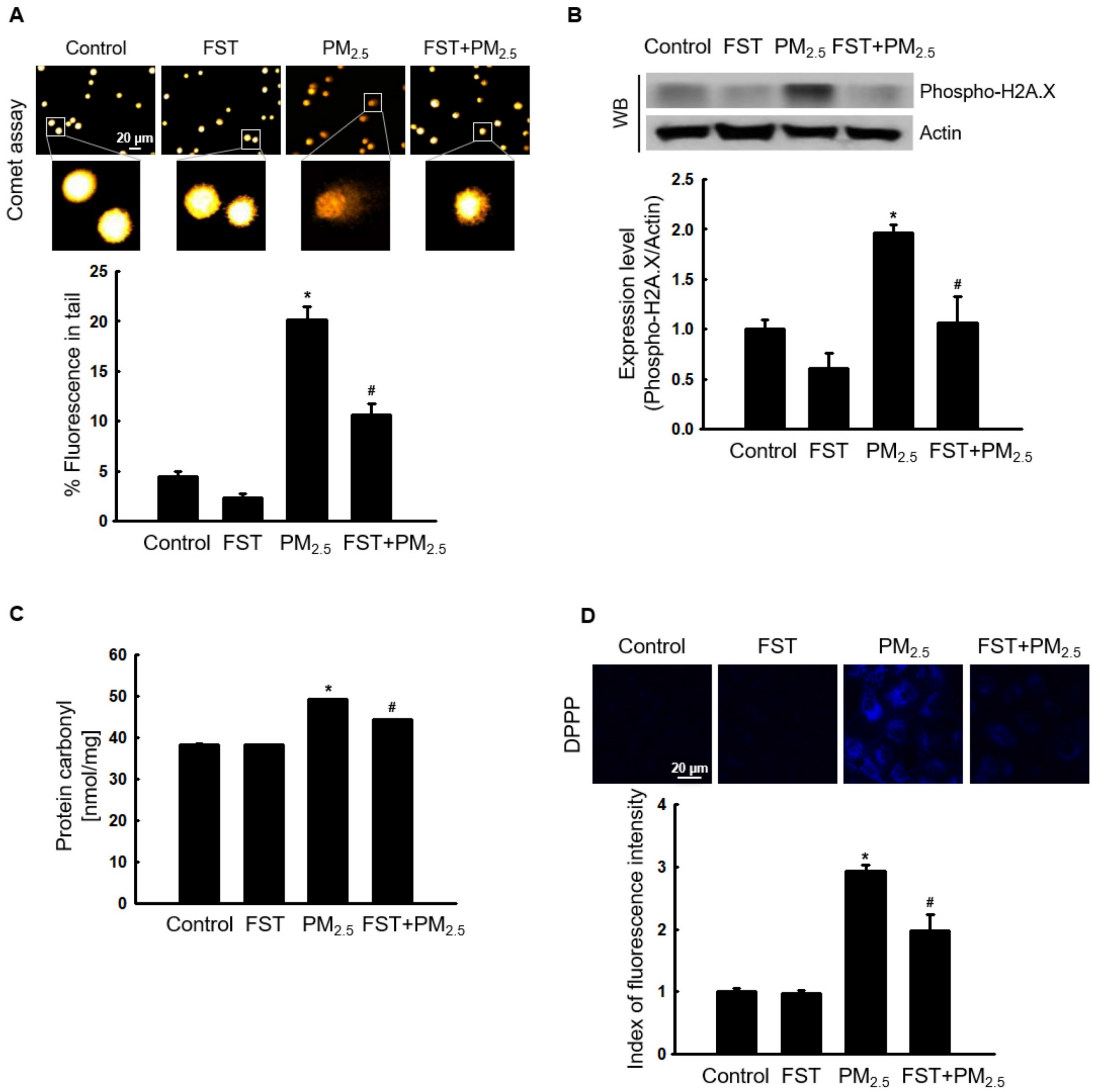
Effect of FST extract on PM_2.5_-induced oxidative damage. (A) DNA breaks were detected by performing an alkaline comet assay. (B) Phosphorylated histone H2A.X protein was detected using western blot assay. Actin was the loading control protein. (C) Protein oxidation was determined by measuring carbonyl formation. (D) Lipid peroxidation was assessed via confocal microscopy after fluorogenic peroxide reactive probe DPPP staining. (A-D) ^*^ p < 0.05 and ^#^* p* < 0.05, compared to the control group and PM_2.5_-treated group, respectively.

Through comet assay, the length of the comet-shaped tail, which indicates the extent of DNA cleavage, significantly increased in cells treated with PM_2.5_. As shown in the fluorescence microscopy image of Figure 2A, the percentage of the cell tail length upon exposure to PM_2.5_ was 20%, whereas the percentage of the tail length for cells pretreated with the FST extract was 11%. In addition, western blot results showed that the phosphorylated histone H2A.X, indicative of double-stranded DNA breaks, was significantly increased after PM_2.5_ treatment, whereas pretreatment with the FST extract reduced it (Figure 2B). The protein carbonyl content was also significantly increased by PM_2.5_ (49 nmol/mg) treatment but significantly decreased by pretreatment of FST extract (44 nmol/mg) (Figure 2C). DPPP oxide fluorescence intensity of lipid peroxidation was enhanced in PM_2.5_-treated cells compared to that in control cells; however, FST extract pretreatment inhibited this increase in the fluorescence intensity, thereby exerting a protective effect on lipid peroxidation caused by PM_2.5_ (Figure 2D).

### Effect of FST extract on PM_2.5_-induced intracellular Ca^2+^ accumulation and apoptosis

**Figure 3:**
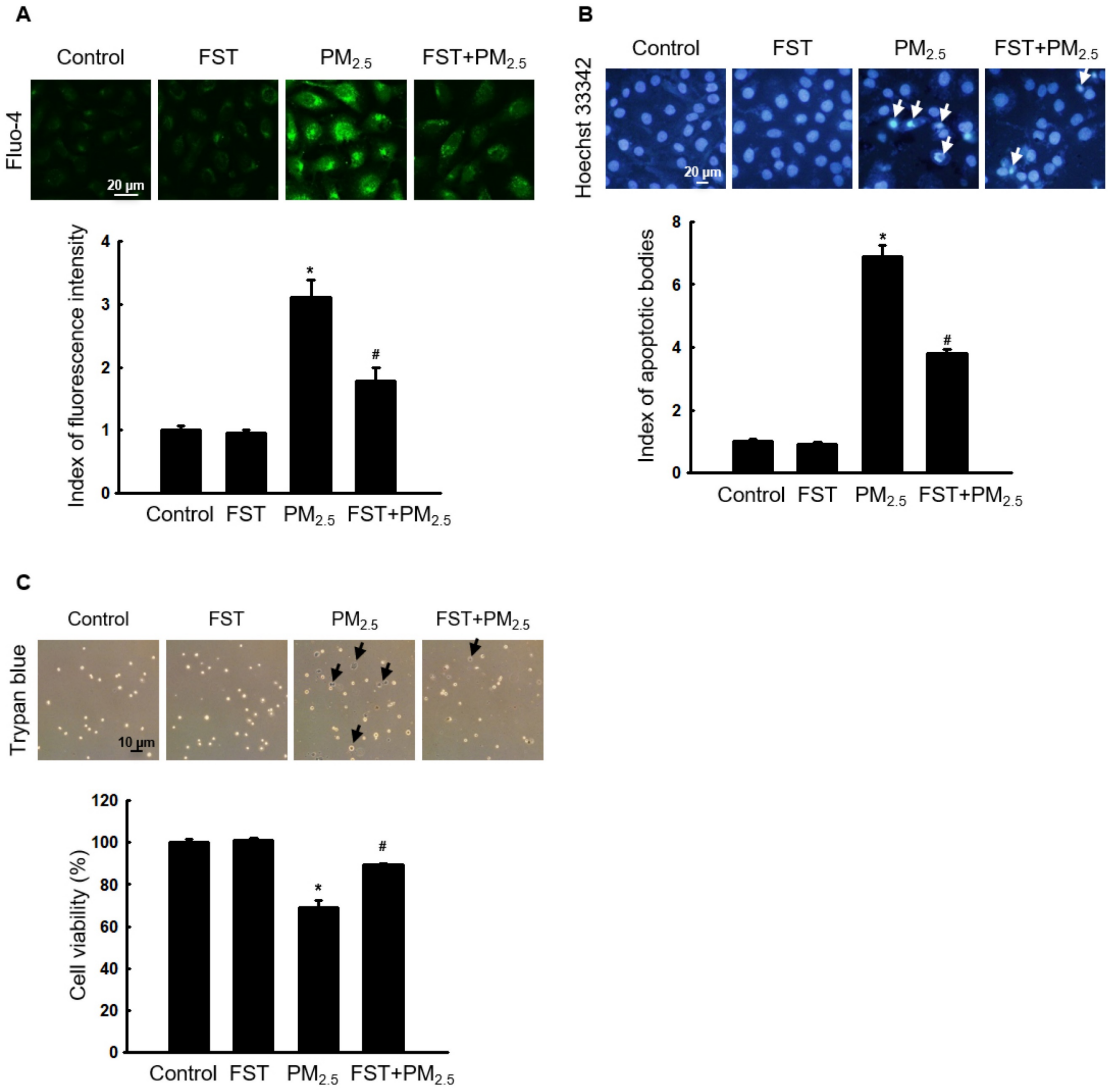
Effect of FST extract on PM_2.5_-induced intracellular Ca^2+^ accumulation and apoptosis. (A) Cells stained with Fluo-4 AM to assess intracellular Ca^2+^ levels were observed using confocal microscopy. (B) Cells stained with Hoechst 33342 dye to detect apoptotic bodies were photographed under fluorescence microscopy, and quantified. Arrows indicate apoptotic bodies. (C) Cell viability was detected using a fluorescence microscope after trypan blue dye staining. (A-C) ^*^ *p* < 0.05 and ^#^ *p* < 0.05, compared to the control group and PM_2.5_-treated group, respectively.

Ca^2+^ signaling mediates oxidative stress-induced ROS production, which disrupts normal physiological pathways and leads to cell death 27. Confocal microscopy data showed that Ca^2+^ fluorescence intensity in PM_2.5_-treated cells was higher than that in control cells. However, pretreatment with the FST extract reduced the PM_2.5_-induced increase in intracellular Ca^2+^ fluorescence intensity (Figure 3A). We previously demonstrated that PM_2.5_ causes cell death by inducing apoptosis in human HaCaT keratinocytes 10. Significant nuclei fragments (apoptotic bodies) were observed in PM_2.5_-treated cells, however, pretreatment with the FST extract decreased them (Figure 3B). In addition, pretreatment with the FST extract in PM_2.5_-treated cells significantly increased 20% of cell viability in PM_2.5_-treated cells (Figure 3C).

### Effect of FST extract on PM_2.5_-induced mitochondrial damage

**Figure 4:**
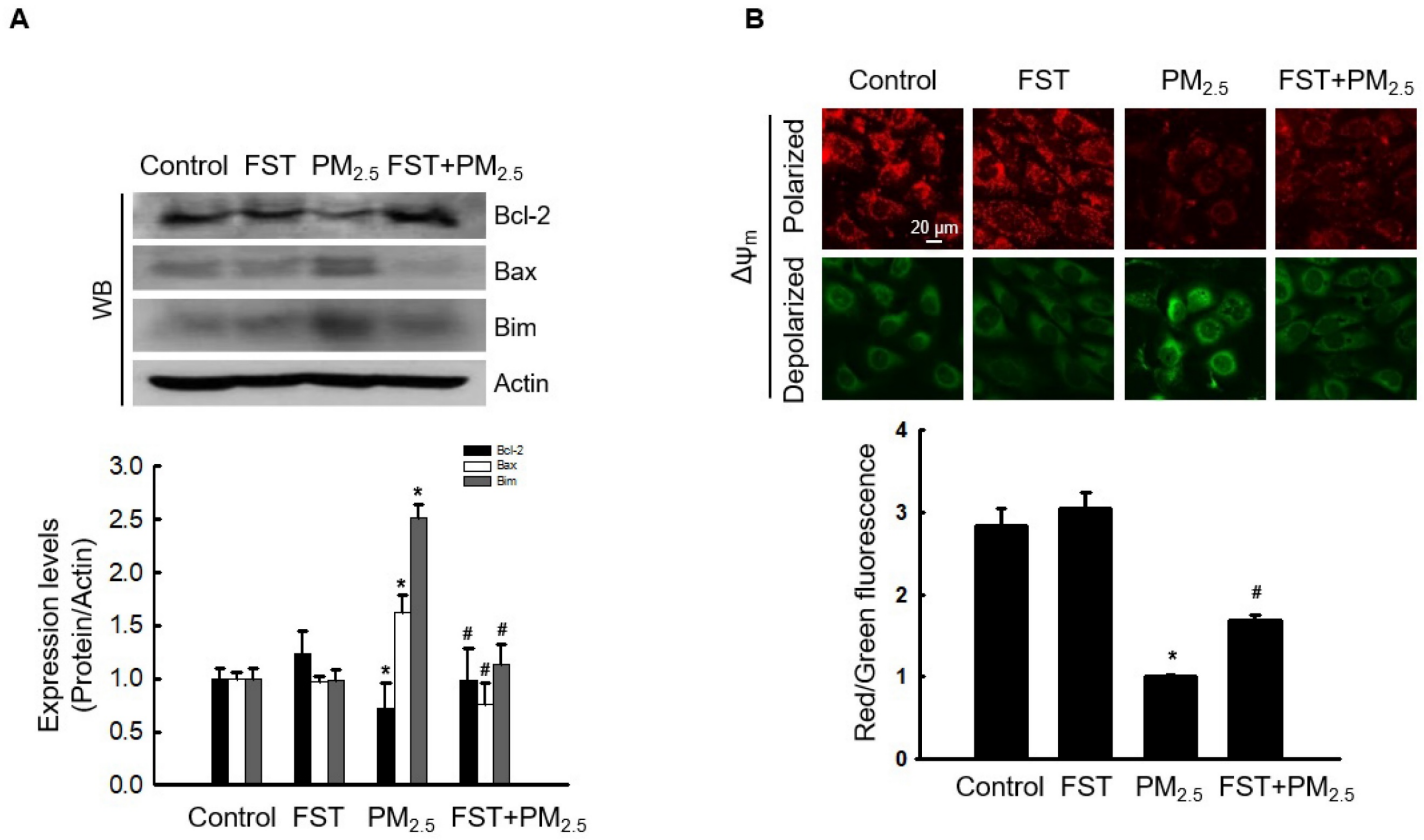
Effect of FST extract on PM_2.5_-induced mitochondrial cell death pathway. (A) Western blot analysis by reacting Bcl-2, Bax, Bim, and actin antibodies was performed. Actin was the loading control protein. (B) Δψ_m_ was evaluated after JC-1 dye staining via confocal microscopy. (A, B) ^*^ *p* < 0.05 and ^#^ *p* < 0.05, compared to the control group and PM_2.5_-treated group, respectively.

Recent studies have indicated that PM_2.5_‑induced apoptosis is mediated by mitochondria 28, 29. We demonstrated that PM_2.5_‑induced oxidative stress promotes mitochondrial damage 10. Bcl-2-family proteins with pro-apoptotic or anti-apoptotic activities regulate the mitochondrial apoptosis pathway by controlling the permeabilization of mitochondrial outer membranes 30. Exposing cells to PM_2.5_ significantly reduced expression of the anti-apoptotic protein Bcl-2, whereas pretreatment with the FST extract significantly prevented this reduction. In contrast, PM_2.5_ increased expression of the pro-apoptotic proteins Bax and Bim, and the FST extract decreased them (Figure 4A). Mitochondria comprise sites of oxidative phosphorylation and ROS production, and they are closely related to the regulation of apoptosis through membrane permeabilization 31. Confocal microscopic images of JC-1 dye staining confirmed that control cells showed strong red fluorescence, forming many JC-1 aggregates, indicating mitochondrial membrane polarization. However, the JC-1 aggregates in the PM_2.5_-treated group were significantly reduced and mostly present in the monomeric form with a significant decrease in red fluorescence intensity and enhanced green fluorescence, indicating mitochondrial membrane depolarization. This phenomenon in PM_2.5_-treated cells was significantly improved by pretreatment with the FST extract (Figure 4B). These results indicate that the FST extract protects against PM_2.5_-induced apoptosis via the mitochondrial pathway.

### Effect of FST extract on PM_2.5_-induced caspase-dependent apoptosis

**Figure 5:**
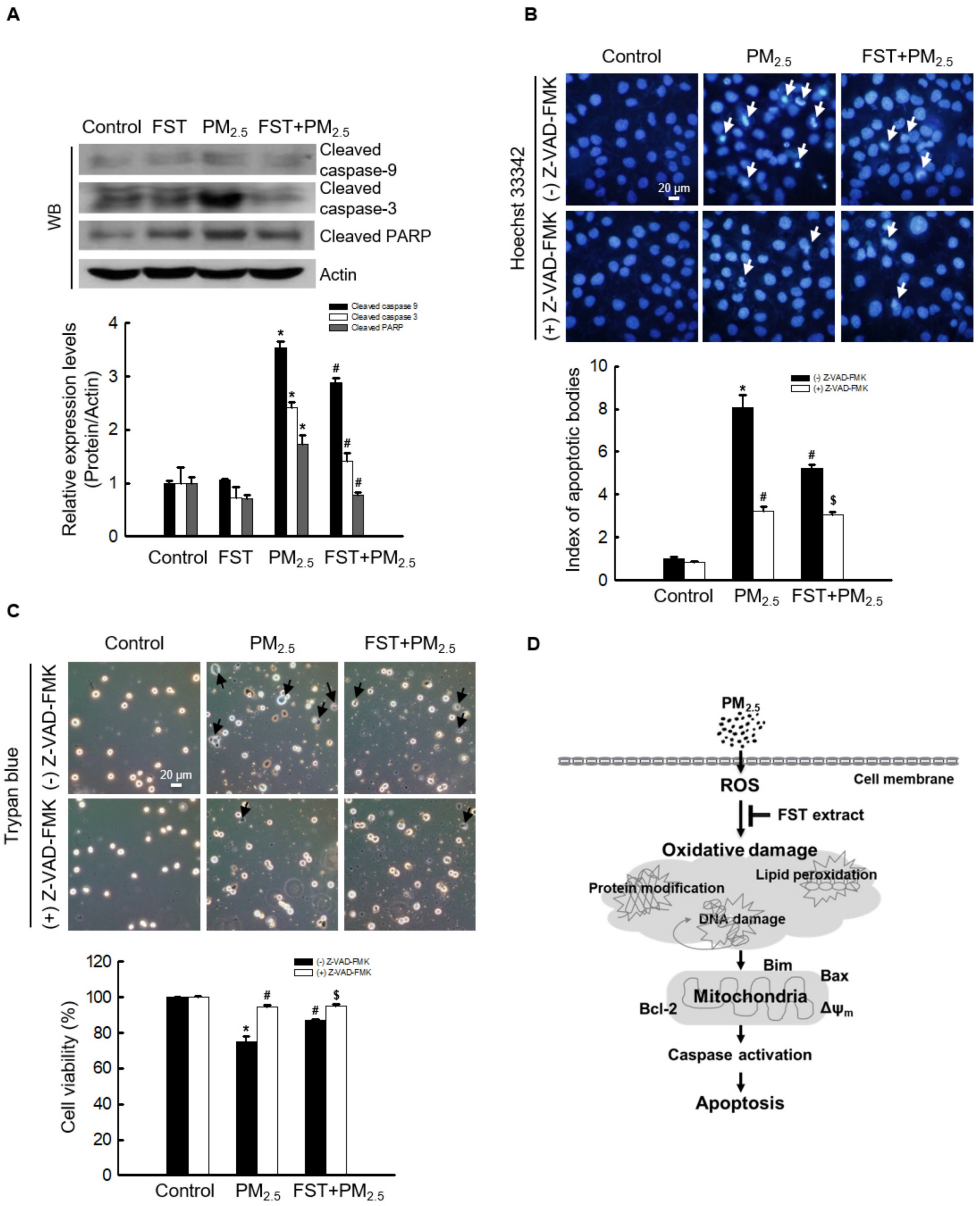
Effect of FST extract on PM_2.5_-induced apoptosis via caspase-dependent signaling. (A) Western blot analysis by reacting caspase-9, caspase-3, PARP, and actin antibodies was assessed. Actin was the loading control protein. (B) After staining with Hoechst 33342 dye, the nuclei were photographed under fluorescence microscopy. The apoptotic bodies were quantified and indicated by the arrows. (C) Cells stained with trypan blue reagent were assessed for detection of cell viability. (A-C) ^*^ *p* < 0.05, ^#^ *p* < 0.05 and ^$^* p* < 0.05, compared to control group, PM_2.5_-treated group and FST extract+PM_2.5_ group, respectively. (D) Schematic diagram of the mechanism underlying the protective effect of FST extract on PM_2.5_-induced cell damage.

The effect of FST extract on the caspase pathway-related proteins was studied. Disruption of the mitochondrial membrane is known to result in the activation of caspase-9 32; thus, the active form of caspase-9 and its target caspase-3 were analyzed via western blotting. The FST extract inhibited the PM_2.5_-induced expression of active caspase-9, active caspase-3, and cleaved PARP (Figure 5A). To determine the FST extract's potential modulation of the caspase-dependent pathway in mitigating PM_2.5_-induced cell damage, we performed the following experiments using the pan-caspase inhibitor Z-VAD-FMK. Fluorescence microscopic observations of nuclei showed intact nuclei in control cells and the fragmented nuclei (apoptotic bodies) in PM_2.5_-treated cells (Figure 5B). However, in the Z-VAD-FMK or FST extract pretreated group, PM_2.5_-induced apoptotic body formation was significantly reduced. Furthermore, the Z-VAD-FMK+FST+PM_2.5_ group increased the protective effect compared to the FST+PM_2.5_ group (Figure 5B). Finally, we studied cell viability using the trypan blue reagent, and the data showed a similar trend (Figure 5C).

## Discussion

Urgent action is needed to address increasing air pollution hazards, including the development of skin-protective agents against PM_2.5_, which has been shown to induce ROS in many studies 3-5. Excessive ROS production is accompanied by the inadequate removal or neutralization of antioxidants by the associated defense system. Excessive ROS in the body can lead to oxidative stress, and their effects vary, not always being harmful 33. Mild oxidative stress protects the body from infections and diseases. For example, studies have found that oxidative stress limits the spread of melanoma cells in mice 34. However, long-term oxidative stress causes oxidative damage to biological macromolecules, such as DNA, proteins, and lipids, which disrupts cell signaling, reduces equivalent cell sources and energy, and subsequently results in cell death 33. This can play an important role in the development of various pathological phenomena or diseases such as inflammation and aging. Recently, we elucidated that PM_2.5_ induces apoptosis in human HaCaT keratinocytes by stimulating oxidative stress, ultimately leading to cell death 10.

In the context of these studies, finding inhibitors that prevent cell damage caused by PM_2.5_ is important. Recently, we demonstrated that active ingredients in marine organisms protect against PM_2.5_-induced subcellular skin dysfunction and skin aging 35, 36. Based on this, to eliminate excessive oxidative stress induced by PM_2.5_, we searched for marine antioxidants, focusing on *L. japonica*, and analyzed the effects of its extract on PM_2.5_-induced cell damage. The active ingredients of *L. japonica,* such as essential amino acids, and essential fatty acids, have significant antioxidant activity 13, 16, and the FST extract through fermentation enhances the activity of endogenous antioxidant enzymes, such as superoxide dismutase, glutathione peroxidase, and glutathione reductase 37. Moreover, the rich GABA component in the FST extract increases antioxidant activity and exerts a prophylactic or therapeutic effect on certain diseases, such as cognitive impairment and liver damage 20, 21, 37. In particular, many studies showing the possibility of using GABA for improving skin aging and moisturizing functions have recently been conducted 23. Therefore, we believe that the effect of the FST extract on PM_2.5_-induced skin cell damage is likely related to its antioxidant activity.

In the current study, we demonstrated that the FST extract scavenges PM_2.5_-induced ROS and protects against damage to macromolecular substances, such as DNA, protein, and lipids, caused by oxidative stress (Figures 1-3). The caspase-dependent apoptotic pathway is a typical pathway underlying apoptosis 38. The Bcl-2 family of proteins includes pro-apoptotic and anti-apoptotic proteins that are activated by a variety of noxious stimuli.

The anti-apoptotic protein Bcl-2 suppresses apoptosis induced by pro-apoptotic Bax and directly inhibits caspase family members, including caspase-3 and caspase-9 39, 40. Bcl-2 protein blocks the oligomerization of Bax and closes the mitochondrial membrane pores 41-43. Meanwhile, the Bim initiates apoptosis by activating the mitochondrial apoptotic effectors Bax and Bak 44. The FST extract was found to inhibit the PM_2.5_-mediated disruption of mitochondrial membrane potential and enhance the expression of Bcl-2 or inhibit the expression of Bax and Bim, resulting in anti-apoptotic and survival effect (Figure 4). We confirmed that the levels of the apoptosis initiator caspase-9 and executor caspase-3 increased dramatically when treated with PM_2.5_, but were significantly suppressed when pretreated with FST extract, confirming that FST extract is involved in the apoptosis signaling pathway caused by PM_2.5_. Additionally, the Z-VAD-FMK+FST+PM_2.5_ group had enhanced protection compared to that of the FST+PM_2.5_ group (Figures 5B and 5C). This result demonstrates the protective effect of the FST extract on PM_2.5_-induced apoptosis through inhibition of the caspase signaling pathway.

The effect of the FST extract is most likely based on the fact that its active ingredient, such as GABA, inhibits intracellular damages mediated by PM_2.5_-induced oxidative stress through its own antioxidant activity (Figure 5D).

## Conclusions

Taken together, FST extract provides valuable information for developing skin care products suitable for environments with severe air pollution.

## Figures and Tables

**Table 1:** Reagent information

Reagent	Action	Company/City/State/Country
Thiazolyl blue tetrazolium bromide (MTT)	Cell viability	Sigma-Aldrich Co./St. Louis/MO/USA
2,2-Diphenyl-1-picrylhydrazyl (DPPH)	Free radical	
2',7'-Dichlorodihydrofluorescein diacetate (H_2_DCFDA)	Cell-permeant ROS indicator	
N-acetylcysteine (NAC)	Antioxidant	
5,5-Dimethyl-1-pyrroline-N-oxide (DMPO)	Spin trap	
Actin	Antibody	
Phospho-histone H2A.X(Ser139)	Primary antibody	Cell Signaling Technology/Beverly/MA/ USA
Caspase-3		
Caspase-9		
Poly ADP ribose polymerase (PARP)		
Bcl-2 (N-19)	Primary antibody	Santa Cruz Biotechnology/Santa Cruz/CA/USA
Bax (B-9)		
Bim (H-191)		
Fluo-4 AM	Cell-permeable Ca^2+^ indicator	ThermoFisher Scientific/MA/USA
Diphenyl-1-pyrenylphosphine (DPPP)	Fluorogenic peroxide reactive probe	
JC-1	Indicator of mitochondrial membrane potential	
Hoechst 33342	Fluorescent nucleic acid stain	ImmunoChemistryTechnologies/Davis/CA/USA
Z-VAD-FMK	Pan caspase inhibitor	TocrisBioscience/Minneapolis/MN/USA

**Table 2:** ESR parameters

Parameters Items	Superoxide anion	Hydroxyl radical
Magnetic field	336 mT	336 mT
Power	5.00 mW	1.00 mW
Frequency	9.4380 GHz	9.4380 GHz
Modulation width	0.2 mT	0.2 mT
Amplitude	500	100
Sweep time	0.5 min	0.5 min
Sweep width	10 mT	10 mT
Time constant	0.03 s	0.03 s
Temperature	25 °C	25 °C
